# Supplementary material for: Problem-solving ability and future time perspective among the Chinese nursing interns: The mediating role of future work self
Source: PLoS One. 2024 Aug 8;19(8):e0308669. doi: 10.1371/journal.pone.0308669 (PMC11309400; doi:10.1371/journal.pone.0308669)
Supplement: S1 Appendix — (DOCX) [file pone.0308669.s003.docx]

**Informed consent form**

Dear nursing interns,

Hope all is well with you!

First of all, thank you very much for taking time out of your busy schedule to fill out our questionnaire. The questionnaire consists of four sections. Please read the following contents carefully. If you have any questions or questions, please ask the researcher carefully.

The aims of this study are (1) to investigate problem-solving ability, future time perspective, and future work self among the Chinese nursing interns, (2) to examine the correlations among problem-solving ability, future time perspective, and future work self, (3) to further explore the mediating role of future work self between problem-solving ability and future time perspective, and (4) to provide a theoretical basis for constructing the interventional measures to improve the problem-solving ability of the Chinese nursing interns.

This study adopts questionnaire survey method. If you agree to participate in the study, please fill in the questionnaire truthfully according to your actual situation and ask the researchers in time if you don't understand the questions.

You voluntarily decide whether to participate in this study, and whether you participate in this study or not, you will not be affected in any way. All the information you provide is only used for this study, and it is kept safely, and will not be disclosed and made public to anyone without your permission. Thank you for your cooperation! During the research, if you have any questions, please feel free to consult the relevant personnel, and we will do our best to help you.

Wish: good health and all the best!

**Signature of researcher:**

**Date:**

The researcher has explained the research related matters to me, and I have made clear the purpose, process and significance of the research. I agree to participate in this research and sign here!

**Signature of participants:**

**Date:**

**1. First section: The Demographic Characteristics Questionnaire (Note: Tick "√" in the box that best suits your situation.)**

| **Characteristics** | **Tick "√"** | **Characteristics** | **Tick "√"** |
| --- | --- | --- | --- |
| **Gender** |  | **Whether is a only child** |  |
| Male | □ | Yes | □ |
| Female | □ | No | □ |
| **Age (years)** |  | **Have been internship time (months)** |  |
| 19～＜22 | □ | 0～＜3 | □ |
| 22～＜24 | □ | 3～＜6 | □ |
| ≥24 | □ | 6～＜9 | □ |
| **Education background** |  | ≥9 | □ |
| Junior college degree | □ | **Number of night shifts per month (times)** |  |
| Bachelor degree | □ | ＜5 | □ |
| Master degree or above | □ | 5～10 | □ |
| **Residence** |  | ＞10 | □ |
| Cities | □ | **Whether are engaged in nursing after graduation** |  |
| Township | □ | Yes | □ |
| Rural areas | □ | No | □ |
| **Character trait** |  | **Academic performance during the school** |  |
| Extroverted | □ | Better (top 1/3 of the grade) | □ |
| Introverted | □ | Intermediate (1/3 of the grade) | □ |
| **Whether is a student cadre** |  | Poor (bottom 1/3 of the grade) | □ |
| Yes | □ | **Satisfaction with clinical teaching** |  |
| No | □ | Dissatisfied | □ |
| **Preference for nursing profession** |  | General | □ |
| Dislike | □ | Satisfied | □ |
| General | □ | **Whether have a plan for future career** |  |
| Like | □ | Yes | □ |
|  |  | No | □ |

**2. Second section: The Social Problem-Solving Inventory (SPSI) (Note: Tick "√" in the box that best suits your situation.)**

| Items | Not in conformity at all | Not in conformity | Uncertain | In conformity | Very in conformity |
| --- | --- | --- | --- | --- | --- |
| **Negative problem orientation** | | | | | |
| 1.I feel panic and fear when I want to solve major problems. | □ | □ | □ | □ | □ |
| 2.I feel nervous and lack confidence when making important decisions. | □ | □ | □ | □ | □ |
| 3.I will feel discouraged when I fail to solve the problem for the first time. | □ | □ | □ | □ | □ |
| 4.The problem makes me feel very distressed. | □ | □ | □ | □ | □ |
| 5.I feel depressed and uncomfortable when there are major problems to be solved. | □ | □ | □ | □ | □ |
| **Rational problem-solving** | | | | | |
| 6.After solving the problem in my own way, I will see if the situation is better than before and think about my feelings at that time in time. | □ | □ | □ | □ | □ |
| 7. After solving the problem in a certain way, I will carefully observe whether the situation is better than before. | □ | □ | □ | □ | □ |
| 8. When making a decision, I will compare and measure the different effects of each choice. | □ | □ | □ | □ | □ |
| 9. When making a decision, I will systematically compare and weigh each choice. | □ | □ | □ | □ | □ |
| 10. When solving problems, I will keep in mind my expected results. |  |  |  |  |  |
| **Positive problem orientation** | | | | | |
| 11.When trying to solve a problem, I usually come up with different solutions and then synthesize a more feasible one. | □ | □ | □ | □ | □ |
| 12.When I failed to solve the problem for the first time, I knew that if I persisted and didn't give up easily, I would eventually find a solution. | □ | □ | □ | □ | □ |
| 13.I believe there is a solution to any problem. | □ | □ | □ | □ | □ |
| 14.When solving problems, I will analyze the situation and try to find out the factors that will hinder my success. | □ | □ | □ | □ | □ |
| 15.When something happens to me, I will try to solve it as soon as possible. | □ | □ | □ | □ | □ |
| **Avoidance style** | | | | | |
| 16.I will delay solving the problems around me. | □ | □ | □ | □ | □ |
| 17.After solving the problem in a certain way, I didn't carefully review the results. | □ | □ | □ | □ | □ |
| 18.I tried my best to avoid the problems that happened to me. | □ | □ | □ | □ | □ |
| 19.I would rather avoid the problem than try to find a solution. | □ | □ | □ | □ | □ |
| 20.I will always put off solving problems, and it is often too late to solve them. | □ | □ | □ | □ | □ |
| 21.I spend more time avoiding problems than solving them. | □ | □ | □ | □ | □ |
| **Impulsive/negligent style** | | | | | |
| 22.When trying to solve a problem, I will act according to the first solution that comes to mind. | □ | □ | □ | □ | □ |
| 23.Do what first comes to mind. | □ | □ | □ | □ | □ |
| 24.When making a decision, I don't spend time thinking about the good and bad of every choice. | □ | □ | □ | □ | □ |
| 25.When making a decision, I will take the attitude of "I want to do it" without carefully considering the consequences of each choice. | □ | □ | □ | □ | □ |

**3. Third section: The Future Time Perspective Inventory (FTPI) (Note: Tick "√" in the box that best suits your situation.)**

| Items | Non-conformance | Only some conformance | Some conformance | Very conformance |
| --- | --- | --- | --- | --- |
| **Long-term target orientation** | | | | |
| 1.I have a goal to work hard every day. | □ | □ | □ | □ |
| 2.I think my future is mainly decided by fate. | □ | □ | □ | □ |
| 3.I often remind myself not to forget the most important goal in the future. | □ | □ | □ | □ |
| 4.I believe that I have the ability to build my own bright tomorrow. | □ | □ | □ | □ |
| 5.Looking ahead, I have a lot of things to do. | □ | □ | □ | □ |
| **Future intentions** | | | | |
| 6.I know what my main task is at present. | □ | □ | □ | □ |
| 7.Once I set my goal, I will take concrete measures to achieve it. | □ | □ | □ | □ |
| 8.I often imagine what I will achieve in five years. | □ | □ | □ | □ |
| 9.I think my future is bright. | □ | □ | □ | □ |
| **Behavioral commitments** |  |  |  |  |
| 10.I evaluate information about life development according to whether it is conducive to achieving my long-term goals. | □ | □ | □ | □ |
| 11.I often imagine how I will change in my later life. | □ | □ | □ | □ |
| 12.Once I have decided what to do, I will consider how to finish what I want to do. | □ | □ | □ | □ |
| 13.I know there are many tasks to be completed in the future. | □ | □ | □ | □ |
| **Future effectiveness** | | | | |
| 14.I finished the plan on time by pushing it forward step by step. | □ | □ | □ | □ |
| 15.I often reflect on what my long-term goal in life is. | □ | □ | □ | □ |
| 16.I am full of confidence in my future. | □ | □ | □ | □ |
| **Awareness of purpose** | | | | |
| 17.The trajectory of my life is determined by forces beyond my control. | □ | □ | □ | □ |
| 18.I often feel that life has no purpose. | □ | □ | □ | □ |
| 19.I am quite concerned about other people's negative comments on my future development. | □ | □ | □ | □ |
| 20.I have a vague idea of my future. | □ | □ | □ | □ |

**4. Fourth section: The Future Work Self Scale (FWSS) (Note: Tick "√" in the box that best suits your situation.)**

| Items | Very disagree | Disagree | A little disagree | Not sure | Agree | A little agree | Very agree |
| --- | --- | --- | --- | --- | --- | --- | --- |
| 1.My future is easy to imagine. | □ | □ | □ | □ | □ | □ | □ |
| 2.I have a very clear psychological picture for the future. | □ | □ | □ | □ | □ | □ | □ |
| 3.I can easily imagine my future job. | □ | □ | □ | □ | □ | □ | □ |
| 4.I know exactly who I want to be and what kind of person I want to be in my future work. | □ | □ | □ | □ | □ | □ | □ |
